# Correlation of MKI67 with prognosis, immune infiltration, and T cell exhaustion in hepatocellular carcinoma

**DOI:** 10.1186/s12876-021-01984-2

**Published:** 2021-11-01

**Authors:** Shi-yi Wu, Pan Liao, Lu-yu Yan, Qian-yi Zhao, Zhao-yu Xie, Jie Dong, Hong-tao Sun

**Affiliations:** 1 Department of Cardiology, Inner Mongolia Forestry General Hospital, Yakeshi, 022150 China; 2 Department of Neurology, Inner Mongolia Forestry General Hospital, Yakeshi, 022150 China; 3 Ophthalmology Department, The Second Xiangya Hospital, Central South University, No. 139, Renmin Middle Road, Changsha, 410000 China; 4 Department of Gastroenterology, Inner Mongolia Forestry General Hospital, Lincheng Road, Yakeshi, 022150 China; 5 Department of Cardiology, Affiliated Hospital of Inner Mongolia University for Nationalities, Tongliao, 028000 China

**Keywords:** Immunotherapy, Immune infiltration, T cells exhaustion, Prognosis, MKI67

## Abstract

**Background:**

MKI67 plays a vital role in the tumour microenvironment (TME) and congenital immunity. The present work focuses on exploring the prognosis prediction performance of MKI67 and its associations with T cell activity and immune infiltration within numerous cancers, especially hepatocellular liver carcinoma (LIHC).

**Methods:**

Oncomine, GEPIA2, and HPA were adopted to analyse MKI67 levels in different types of cancers. The prognostic prediction performance of MKI67 was evaluated through the TCGA portal, GEPIA2, LOGpc, and Kaplan–Meier Plotter databases. The associations of MKI67 with related gene marker sets and immune infiltration were inspected through TISIDB, GEPIA2, and TIMER. We chose MKI67 to analyse biological processes (BPs) and KEGG pathways related to the coexpressed genes. Furthermore, the gene–miRNA interaction network for MKI67 in liver cancer was also examined based on the miRWalk database.

**Results:**

MKI67 expression decreased in many cancers related to the dismal prognostic outcome of LIHC. We found that MKI67 significantly affected the prognosis of LIHC in terms of histology and grade. Increased MKI67 levels were directly proportional to the increased immune infiltration degrees of numerous immune cells and functional T cells, such as exhausted T cells. In addition, several critical genes related to exhausted T cells, including TIM-3, TIGIT, PD-1, LAG3, and CXCL13, were strongly related to MKI67. Further analyses showed that MKI67 was associated with adaptive immunity, cell adhesion molecules (CAMs), and chemokine/immune response signal transduction pathways.

**Conclusion:**

MKI67 acts as a prognostic prediction biomarker in several cancers, particularly LIHC. Upregulation of MKI67 elevates the degree of immune infiltration of many immune cell subtypes, including functional T cells, CD4+ T cells, and CD8+ T cells. Furthermore, MKI67 shows a close correlation with T cell exhaustion, which plays a vital role in promoting T cell exhaustion within LIHC. Detection of the MKI67 level contributes to prognosis prediction and MKI67 modulation within exhausted T cells, thus providing a new method to optimize the efficacy of anti-LIHC immunotherapy.

**Supplementary Information:**

The online version contains supplementary material available at 10.1186/s12876-021-01984-2.

## Introduction

Liver hepatocellular carcinoma (LIHC), a frequently occurring liver cancer, affects 6/100,000 people every year and is a primary cause of cancer-associated mortality [1, 2]. Current targeted drug therapy cannot provide a satisfactory therapeutic effect because of several diverse factors, including LIHC drug resistance, biological behaviour, and clinical factors [3]. The uncertainty of the molecular mechanisms related to cancer genesis and development further complicates efficient anti-LIHC treatment [4]. In addition, the lack of disease stage- or tumour type-specific markers has dramatically hindered the prevention and management of LIHC. In this regard, it is necessary to elucidate the tumour-immune interaction phenotypes and identify new immune-associated therapeutic targets for liver cancers.

MKi67 expression is found within proliferating cells alone under general conditions [5]. Under normal conditions, MKi67 shows cortical nucleolar localization during interphase and is recruited into condensed chromosomes in mitosis [6, 7]. The MKi67 gene located on chromosome 10q25-ter mainly functions to encode 2 MKi67 isoforms (345 and 395 kDa, respectively) [8–10]. Additionally, the MKi67 level is elevated from the G1 phase to mitosis and shows a rapid decrease later. MKi67 protein expression can be evaluated within cell nuclei at the G1/S/G2 phase and mitosis rather than within quiescent cell nuclei at the G0 phase [11, 12]. As a result, MKi67 expression represents cell proliferation status. Ki67 shows high expression within cancer cells and can be regarded as a prognostic prediction factor for cancer [13, 14]. Furthermore, MKi67 has been studied extensively in retrospective articles as a candidate prognostic prediction factor for cancer proliferation [15, 16]. Plenty of evidence supports the role of MKi67 in diagnosing cancer [17–20]. Cancer cells show high MK167 protein expression, and the positive MKI67 rate (referred to as the labelling index) is related to the clinicopathological characteristics and survival of diverse cancers, such as LIHC [21]. In an article enrolling LIHC cases receiving surgery, high MK167 expression was identified in cancer tissues, which predicted greater tumour grade and early cancer relapse [22, 23]. Furthermore, p53 (encoded via a tumour suppressor gene) and MK167 staining have been extensively adopted to predict LIHC survival postoperatively or even after liver transplantation [24, 25]. The above results indicate that MKI67 plays a vital role in cancer migration, invasion, and progression.

The present work focuses on detecting MKI67 levels and mutations in LIHC cases derived from publicly accessible databases such as The Cancer Genome Atlas (TCGA). MKI67-related functional networks and genomic alterations within LIHC were evaluated based on multidimensional analysis, where the function of MKI67 in tumour immunity was also explored. The findings of this work can help identify novel diagnostic and therapeutic targets for LIHC.

## Materials and methods

### Analysis based on oncomine database

Oncomine is an integrative database covering 86,733 samples and 715 gene expression profiling datasets developed to facilitate data mining [26]. It is utilized in the present work to assess MKI67 levels and patient prognosis in different types of cancers. (https://www.oncomine.org/resource/login.html).

### Analysis based on TIMER database

Tumour Immune Estimation Resource (TIMER, cistrome. shinyapps.io/timer), a kind of easy-to-use web interface, has provided a computational approach for oncology investigators to comprehensively and dynamically analyse and monitor cancer genomic and immunologic data [27]. It contains gene expression profiling data of 10,897 samples covering 32 different kinds of TCGA-derived cancers to estimate six tumour-infiltrating immune cells (TIICs), including CD4+ T cells, CD8+ T cells, dendritic cells (DCs), B cells, neutrophils, and macrophages. This study adopted constrained least-squares fitting for specific gene levels, which negatively correlated with the tumour purity of all cancers [28], for predicting the 6 TIIC subpopulation abundances. Furthermore, “Gene module” and “Diff Exp module” were utilized to analyse the MKI67 level within diverse cancers, as well as the associations of MKI67 level with 6 TIIC subpopulation abundances. The Wilcoxon test assessed the significant difference in MKI67 expression levels. We adopted statistical significance and purity-adjusted partial Spearman’s correlation to evaluate the association between MKI67 levels and immune infiltration. Tumour infiltration degrees for MKI67 across different cancers showing distinct somatic copy number alterations (SCNA) were compared using the “SCNA module” defined by GISTIC 2.0. Typically, the module consists of high amplification (2), arm-level gain (1), diploid/normal (0), arm-level deletion (− 1), and deep deletion (− 2) [29]. Furthermore, we utilized the “Correlation module” to explore the associations of MKI67 levels with TIIC gene markers carefully chosen based on previously published articles, including markers for T cells, B cells, effector T cells, CD8+ T cells, central memory T cells, effector memory T cells, exhausted T cells, resident memory T cells, effector Treg cells, resting Treg cells, neutrophils, T-helper 1 (Th1), dendritic cells (DCs), macrophages, mast cells, and natural killer cells (NK cells) [30–33]. We designed scatterplots of MKI67 gene expression in specific cancers using this module based on statistical significance and Spearman’s correlation analysis. We also displayed gene expression data in the form of a log2 RSEM (RNA-Seq by Expectation–Maximization).

### Analysis based on OnCoLnc database

OncoLnc covers the survival data for 21 TCGA-derived cancers along with the corresponding MiTranscriptome, mRNA, and miRNA data http://www.oncolnc.org/. The cases can be classified into different groups based on gene expression to obtain results using this database. OncoLnc contributes to viewing the Kaplan–Meier plot results for at least one cancer simultaneously. It offers Cox regression data and allows the extraction of sufficient data for analysis. In addition, users can also examine the prognostic significance of the tested genes within 21 cancers simultaneously, facilitating the investigation of the vital functions of specific genes in cancer survival.

### Analysis based on GEPIA2 database

The Gene Expression Profiling Interactive Analysis 2 database (GEPIA2, http://gepia2.cancer-pku.cn/) represents a web-based approach to investigate gene expression and interactions in cancer tissues and noncarcinoma tissues based on Genotype-Tissue Expression (GTEx) and TCGA-derived data, which can further provide customizable functions, such as profiling plotting, differential expression analysis, patient survival analysis, dimensionality reduction analysis, correlation analysis, and similar gene identification. [34] This study adopted “survival analysis” to examine the association of MKI67 levels with the survival of diverse TCGA-derived cancers. In addition, we used Spearman’s correlation analysis to examine the association of MKI67 with the TIIC gene. Both cancer and noncarcinoma sample datasets were used in subsequent analyses.

### Analysis based on Kaplan–Meier plotter database

The Kaplan–Meier Plotter database was developed as an online approach for rapidly assessing the influence of gene expression on 21 cancer survivors, as well as the four significant datasets, namely, breast cancer (BC, n = 6234), lung cancer (LC, n = 3452), ovarian cancer (n = 2190), and gastric cancer (GC, n = 1440) [35]. It was adopted to evaluate the associations of MKI67 levels with the survival of these four cancers. A pan-cancer dataset was used to study MKI67 levels within diverse LIHC subtypes. Meanwhile, we determined HRs (95% CIs) and log-rank P values and plotted the survival curves (http://kmplot.com/).

### TISIDB database analysis

The TISIDB database covers 988 immune-associated anticancer genes reported in data from previous studies, noncarcinoma multiomics data, molecular profiling data, high-throughput screening (HTS) technologies, and different immunological data resources collected based on seven publicly accessible databases [36]. It analyses the associations between the screened genes and chemokines, lymphocytes, and immunomodulators. The present work adopted TISIDB to assess the associations of Annexin levels with LIHC clinical stages and investigate the relationships of MKI67 levels with immunomodulators and lymphocytes (http://cis.hku.hk/TISIDB).

### Analysis based on human protein atlas database

The HPA (https://www.proteinatlas.org/) database covers all pathological and gene expression data collected from numerous studies conducted using diverse cell lines and tissue types [37]. This database was implemented in the present work to examine MKI67 levels within diverse tissues along with MKI67 localization in cells. The direct links to these images according to the human protein atlas are as follows.

### MEXPRESS database analysis

MEXPRESS (https://mexpress.be/) represents a way to visualize data related to DNA methylation status, TCGA expression, clinical information, and the underlying associations [38]. Here, we utilized MEXPRESS to investigate the SPC25 gene methylation status and the association of SPC25 mRNA levels with various clinical features among BC cases.

### Analysis based on LinkedOmics database

LinkedOmics signifies an openly accessible database covering multiomics data of 32 TCGA-derived cancers [39]. We conducted Pearson’s test for statistical analyses of MKI67 coexpression by LinkedOmics of “LinkFinder.” The data are presented as heat map/volcano map/scatter plots. In addition, we utilized LinkedOmics of the “LinkInterpreter” module for Gene Ontology (GO, Biological Process (BP)) annotation, Kyoto Encyclopedia of Genes and Genomes (KEGG) for pathway analysis, and Gene Set Enrichment Analysis (GSEA) for transcription factor-target/miRNA-target/kinase-target enrichments using the threshold of false discovery rate (FDR) < 0.05 for 1000 iterations (http://www.linkedomics.org).

### Analysis based on miRWalk database

Target genes. MiRNAs screened through the miRWalk approaches were enrolled as possible MKI67-regulating miRNAs [40]. (http://mirwalk.umm.uni-heidelberg.de/).

### Statistical methods

The Oncomine database-derived data was presented as ranking, fold-change (FC), and P-values. Survival curves were plotted from TCGA portal, GEPIA2, KM Plotter, LoGPC, and TIMER, (Cox) P-value and HR by the log-rank test. Wilcoxon rank-sum test (two-sided) was used for comparing the infiltration degree of every SCNA category with normal tissue. Spearman correlation was also adopted to assess the association between MKI67 level and other gene or immune infiltration levels in specific cancer types. *P* ≤ 0.05 indicated statistical significance, shown in the figures.

## Results

### MKI67 mRNA expression in diverse human cancers

**Fig. 1:**
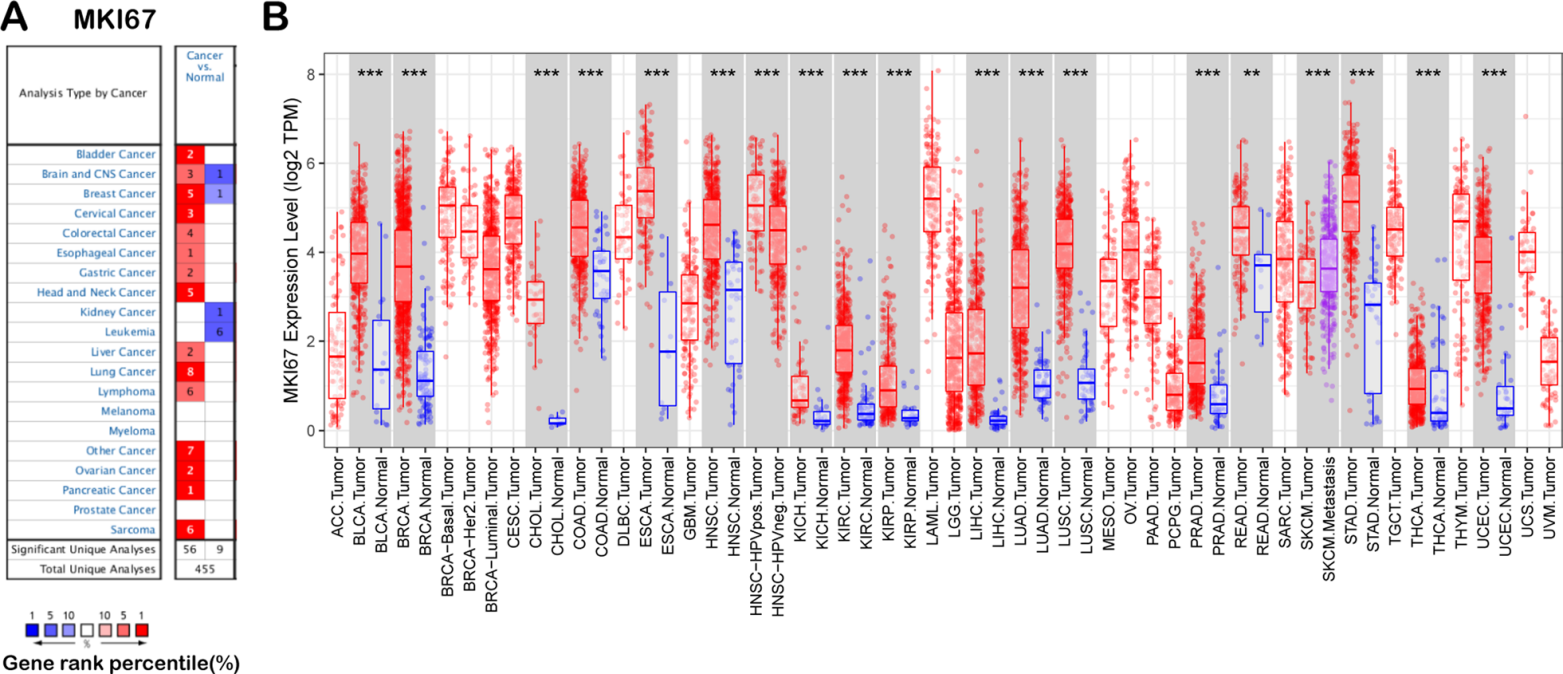
MKI67 expression in diverse human cancer types. **a** MKI67 up-regulation or down-regulation in diverse cancer datasets relative to non-carcinoma samples obtained from Oncomine database. **b** MKI67 expression in diverse TCGA-derived cancers measured using TIMER

We measured MKI67 mRNA expression in tumour and noncarcinoma samples from distinct cancers based on the Oncomine database to determine the differential MKI67 expression between cancer and noncarcinoma samples. According to our results, MKI67 expression was increased in bladder cancer, CNS and brain cancers, breast cancer (BC), head and neck cancer (HNC), colorectal cancer (CRC), oesophageal cancer (EC), cervical cancer, gastric cancer (GC), liver cancer, lung cancer (LC), lymphoma, ovarian cancer, pancreatic cancer, and sarcoma compared with noncarcinoma samples (Fig. 1a). In addition, other datasets also showed decreased levels of MKI67 mRNA expression in CNS and brain cancers and BC, kidney, and leukaemia cancers. Additional file 1: Table S1 provides more data about MKI67 levels in diverse cancers. To better evaluate MKI67 levels in human cancers, we analysed MKI67 levels in various TCGA-derived cancers based on RNA-seq data. Figure 1b shows MKI67 levels within cancer and noncarcinoma samples in TCGA-derived cancers. MKI67 levels decreased significantly in skin cutaneous melanoma (SKCM) relative to matched noncarcinoma samples. However, MKI67 levels increased within BRCA (invasive breast carcinoma), BLCA (urothelial bladder carcinoma), COAD (colon adenocarcinoma), CHOL (cholangiocarcinoma), HNSC (head and neck cancer), ESCA (oesophageal carcinoma), KIRP (kidney renal papillary cell carcinoma), KIRC (kidney renal clear cell carcinoma), KICH (kidney chromophobe), LIHC (hepatocellular liver carcinoma), PRAD (prostate adenocarcinoma), LUSC (lung squamous cell carcinoma), LUAD (lung adenocarcinoma), STAD (stomach adenocarcinoma), READ (rectum adenocarcinoma), UCEC (uterine corpus endometrial carcinoma) and THCA (thyroid carcinoma) relative to noncarcinoma samples.

### Prognosis prediction performance of MKI67 in cancers

To assess the prognosis prediction performance of LIHC in cancer, the associations between the LIHC level and cancer survival were determined from three large-scale cancer databases containing a variety of samples. Typically, this study first determined the impact of LIHC expression on cancer prognostic outcome based on the OncoLnc database. Conspicuously, the LIHC level was markedly associated with the prognosis of eight cancer types: KIRC, BRCA, LGG, KIRP, LUAD, LIHC, SKCM, and PAAD (Table 1).

**Table 1:** Relation between MKI67 expression and patient prognosis of different cancer in OncoLnc database

Cancer	Cox	*P* value	FDR	Rank	Median	Mean
BLCA	0.017	8.30e−01	9.25e−01	14,656	2546.83	2892.19
BRCA	0.196	3.00e−02	3.18e−01	1558	1701.38	2293.89
CESC	0.007	9.60e−01	9.86e−01	15,876	4153.29	4477.01
COAD	− 0.036	7.20e−01	9.11e−01	12,906	3605.44	3927.73
ESCA	− 0.154	2.20e−01	9.72e−01	3737	5406.79	6246.63
GBM	0.051	5.70e−01	9.34e−01	10,197	809.93	1006.12
HNSC	0.037	6.10e−01	8.42e−01	12,004	3773.88	4310.74
KIRC	0.211	1.30e−02	3.84e−02	5613	388.75	544.72
KIRP	0.996	7.80e−09	2.07e−06	62	140.49	283.54
LAML	− 0.011	9.20e−01	9.76e−01	14,335	4542.33	5680.33
LGG	0.249	1.10e−02	2.62e−02	7023	289.2	551.11
LIHC	0.382	4.50e−05	4.76e−03	148	624.13	964.54
LUAD	0.281	2.30e−04	1.34e−02	288	1309.51	1749.87
LUSC	− 0.027	7.00e−01	9.33e−01	12,679	2607.62	2905.06
OV	0.069	3.40e−01	8.37e−01	6799	2414.05	2886.55
PAAD	0.358	1.30e−03	2.86e−02	772	1145.59	1363.72
READ	− 0.073	7.70e−01	9.86e−01	12,829	3665.46	3858.49
SARC	0.157	1.10e−01	3.52e−01	4966	2138.02	2519.99
SKCM	0.172	1.10e−02	6.66e−02	2623	1768.63	2055.23
STAD	− 0.151	7.40e−02	3.95e−01	3156	5150.04	5609.92
UCEC	0.06	6.10e−01	9.95e−01	10,064	2149.23	2492.38

Significantly different results are displayed in this table (cox *P* < 0.05)

**Fig. 2:**
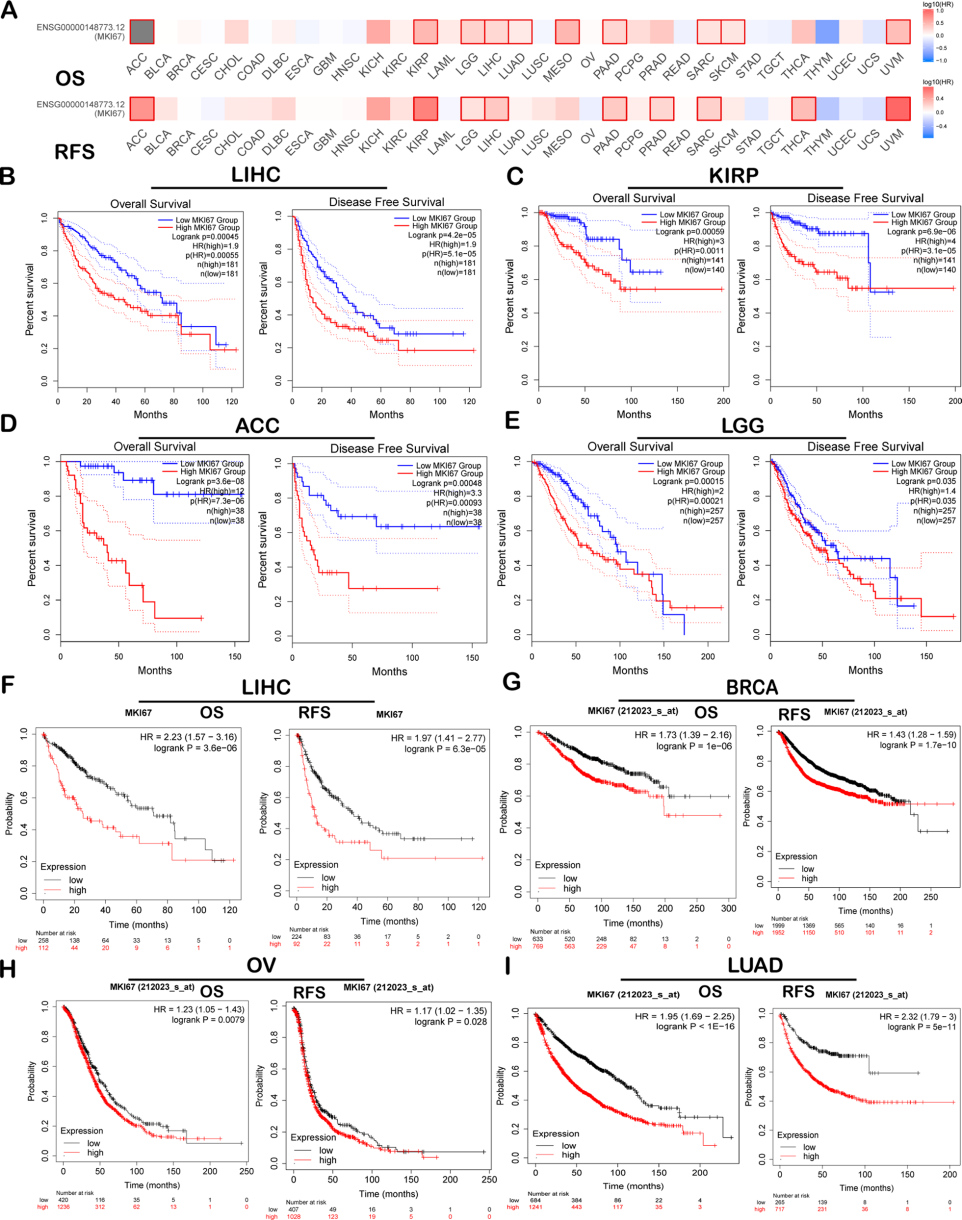
Prognosis prediction performance of MKI in diverse cancers measured using GEPIA2 (**a–e**) and Kaplan–Meier Plot (**f–i**). **a** Survival heatmap for MKI in 33 TCGA-derived cancers. Heatmap showing log10 HRs for MKI67. Blue and red blocks represent decreased and increased risks, separately. Rectangles with frames indicate the significance upon prognosis analysis. DFS and OS curves for **b** LIHC (n = 362), **c** KIRP (n = 281), **d** ACC (n = 76), **e** LGG (n = 514). OS and PFS survival curves in **f** liver cancer (n = 364, n = 316), **g** BC (n = 1402, n = 3951), **h** ovarian cancer (n = 1656, n = 1435), and **i** lung cancer (n = 1925, n = 982). *OS* overall survival, *RFS* relapse-free survival, *DFS* disease-free survival, *PFS *progression-free survival

We conducted comprehensive analysis on the associations of MKI67 level with patient survival from 3 large-scale cancer databases consisting of sufficient samples to assess the prognosis prediction performance of MKI67 for cancer. Figure 2a illustrates the influence of MKI67 level on survival in cancer. The poor overall survival (OS) and disease-free survival (DFS) of LIHC was significantly related to higher MKI67 levels (*P* = 4.5e−4, HR = 1.9; *P* = 4.2e−5, HR = 1.9, respectively) (Fig. 2b). MKI67 upregulation predicted the survival of KIRP (OS: *P* = 0.00059, HR = 3); PFS: *P* = 6.9e−06, HR = 4); ACC (OS: (*P* = 3.6e−08, HR = 12); PFS: *P* = 0.00048, HR = 3.3); LGG (OS: *P* = 0.00015, HR = 2); PFS: *P* = 0.035, HR = 1.4) (Fig. 2c–e). Poor prognosis was also correlated with higher MKI67 expression in PAAD (OS: *P* = 6.7e−05, HR = 2.28); PFS: *P* = 0.014, HR = 2.85); SARC (OS: *P* = 0.0028, HR = 1.82); PFS: *P* = 0.0025, HR = 1.82); and STAD (OS: *P* = 0.01, HR = 0.65); PFS: *P* = 0.0042, HR = 0.39) (Additional file 1: Figure S1F–H). Nonetheless, poor prognosis in ESCA was associated with lower MKI67 expression (OS: *P* = 0.011, HR = 0.18; PFS: *P* = 0.045, HR = 0.37) (Additional file 1: Figure S1F). Furthermore, the level of MKI67 expression significantly differed in disease-free survival and overall survival with PADD (pancreatic adenocarcinoma), SARC (sarcoma), and UVM (uveal melanoma) (Additional file 1: Figure S2A–C). Thereafter, we examined the association of MKI67 level with patient survival from 4 large-scale cancer datasets (including BC, LC, liver cancer and ovarian cancer) based on Kaplan–Meier Plotter. Likewise, MKI67 upregulation predicted the dismal prognostic outcome of LIHC (OS: *P* = 0.00011, HR = 1.96 [1.38–2.77]; PFS: *P* = 1.1e−05, HR = 2.14 [1.51–3.02]), BRCA (OS: *P* = 1e−06, HR = 1.73 [1.39–2.16]; PFS: *P* = 1.7e−10, HR = 1.43 [1.28–1.59]), OV (OS: *P* = 0.0079, HR = 1.23 [1.05–1.43]; PFS: *P* = 0.028, HR = 1.17 [1.02–1.35]), and LUAD (OS: *P* < 1e−16, HR = 1.95 [1.69–2.25]; PFS: *P* = 5e−11, HR = 2.32 [1.79–3]) (Fig. 2f–i).

### Effect of MKI67 on regulating immune molecules

**Fig. 3:**
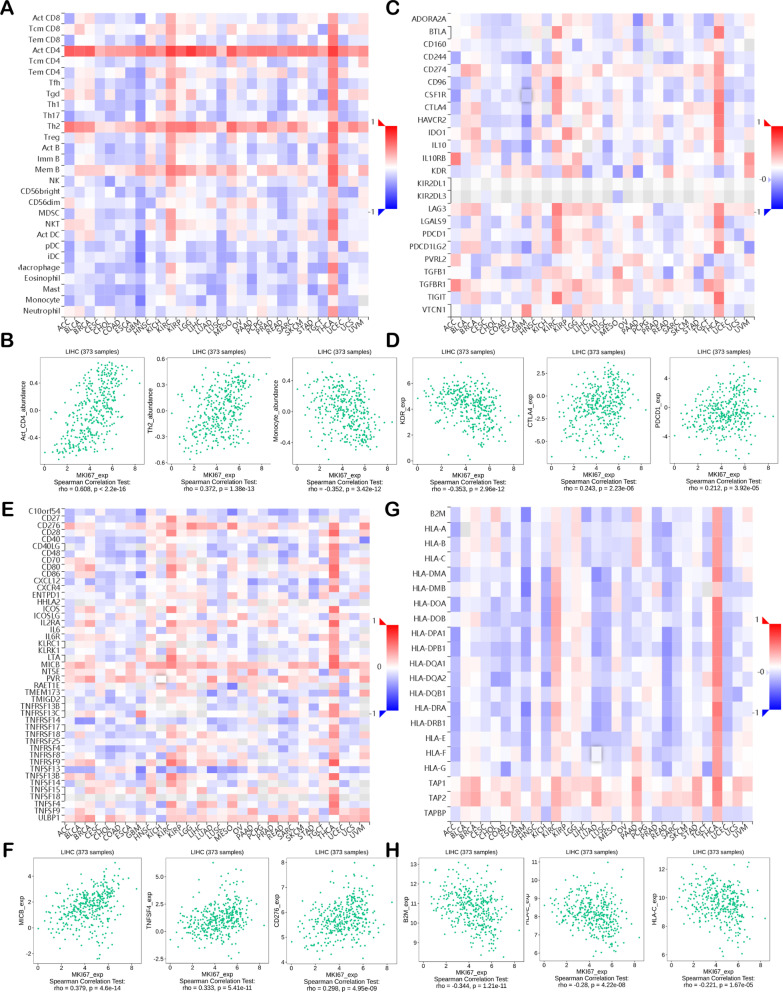
Spearman’s correlation between MKI67 and immunomodulators and lymphocytes (TISIDB). **a** Association of TILs abundances with MKI67 level; **b** the three most significant TILs with the highest Spearman’s correlation with MKI67 levels; **c** association of increasing immune inhibitors with MKI67 level; **d** the three most significant immune inhibitors with the maximum Spearman’s correlation with MKI67 level; **e** association of immunostimulators abundances with MKI67 level; **f** the four most significant immunostimulators with the maximum Spearman’s correlation with MKI67 levels; **g** association of MHC molecules with MKI67 level; **h** the three most significant MHC molecules with the highest Spearman’s correlation with MKI67 level. Blue and red cells stand for negative and positive correlations, separately. Color intensity is directly proportional to correlation strength. *MHC* major histocompatibility complex, *TILs* tumor-infiltrating lymphocytes

Here, we examined Spearman’s correlations of MKI67 levels with immunomodulators based on the TISIDB database (Fig. 3). Figure 3a displays the association of MKI67 levels with TILs. The most significantly correlated lymphocytes were activated CD4 T cells (Act-cd4; Spearman: ρ = 0.608, *P* < 2.2e−16), type 2 helper cells (Th2; Spearman: ρ = 0.372, *P* = 1.38e−13), and monocytes (monocytes; Spearman: ρ = -0.352, *P* = 3.42e−12) (Fig. 3b). Immunomodulators are divided into major histocompatibility complex (MHC) molecules, immune inhibitors, and immunostimulators. Figure 3c reveals the association of MKI67 expression with immunoinhibitors, among which the most significantly correlated ones were KDR (Spearman: ρ = -0.353, *P* = 2.96e−12), PDCD1 (Spearman: ρ = 0.212, *P* = 3.92e−05) and CTLA4 (Spearman: ρ = 0.243, *P* = 2.23e−06) (Fig. 3d). Figure 3e displays the association of MKI67 levels with immunostimulators, where the most significantly correlated ones were MICB (Spearman: ρ = 0.379, *P* = 4.6e−14), CD276 (Spearman: ρ = 0.298, *P* = 4.95e−09), and TNFSF4 (Spearman: ρ = 0.333, *P* = 5.41e−11) (Fig. 3f). Figure 3g represents the association of MKI67 levels with MHC molecules, among which the most significantly correlated are B2M (Spearman: ρ = 0.344, *P* = 1.21e−11), HLA-C (Spearman: ρ = 0.221, *P* = 1.67e−05), and HLA-E (Spearman: ρ = -0.28, *P* = 4.22e−08) (Fig. 3h). Therefore, MKI67 is perhaps related to the regulation of these immune molecules.

### MKI67 is related to immune infiltration degrees within LIHC

**Fig. 4:**
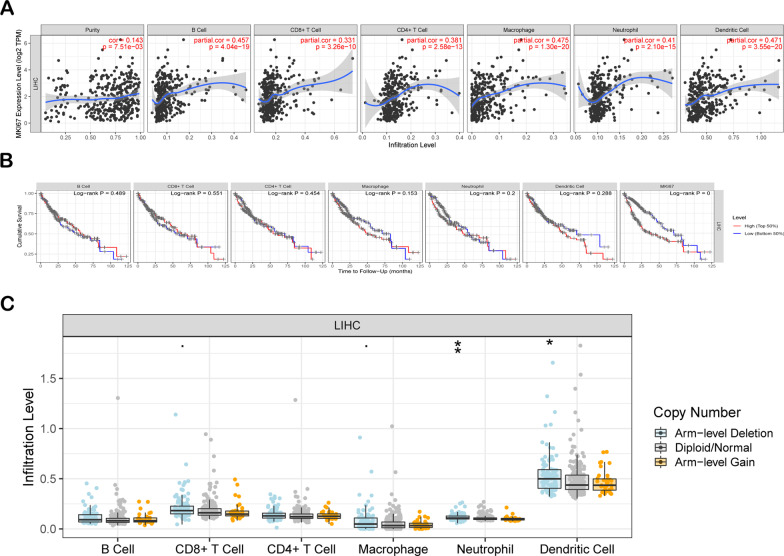
Association of MKI67 with immune infiltration degrees within LIHC. **a** Association of MKI67 level with immune infiltration degrees of CD8+ T cells, CD4+ T cells, B cells, neutrophils, macrophages, DCs, and tumor purity; **b** Kaplan–Meier plots concerning immune infiltration and MKI67 expression within LIHC; **c** tumor-infiltration degrees of LIHC displaying diverse SCNAs defined using GISTIC 2.0, which include high amplification (2), arm-level gain (1), diploid/normal (0), arm-level deletion (− 1), and deep deletion (− 2)

We can estimate lymph node metastasis (LNM) and the survival of cancer cases separately based on lymphocyte infiltration degrees within a tumour [41–43]. Therefore, we further analysed the association of MKI67 levels with immune cell infiltration degrees within 39 cancers based on the TIMER database (Additional file 1: Figure S2). Consequently, MKI67 levels were significantly related to tumour purity within 14 cancers and B cell infiltration degrees within 23 cancers. MKI67 was also related to CD4+ T cell infiltration degrees in 22 cancers, CD8+ T cell infiltration degrees in 19 cancers, DC infiltration degrees in 25 cancers, neutrophil infiltration degrees in 24 cancers, and macrophage infiltration degrees in 17 cancers. However, MKI67 expression was not related to the infiltration degrees of CD4+ T cells, CD8+ T cells, B cells, macrophages, DCs, or neutrophils within cholangiocarcinoma (CHOL) (Additional file 1: Figure S2H). In LIHC, MKI67 expression indicated an increase in the infiltration degrees of CD4+ T cells (R = 0.381, *P* = 2.58e−13), CD8+ T cells (R = 0.331, *P* = 3.26e−10), DCs (R = 0.471, *P* = 3.55e−20), neutrophils (R = 0.41, *P* = 2.10e−15), and macrophages (R = 0.475, *P* = 1.30e−20) (Fig. 4a). Furthermore, in some types of cancers, including THCA, BRCA, SKCM, KIRC, and GBM, the immune infiltration degrees were markedly associated with MKI67 (Additional file 1: Figure S2). This study also presented Kaplan–Meier plots based on the TIMER database to explore the association of immune cell infiltration degrees with MKI67 expression in LIHC. Hence, MKI67 levels were not significantly related to the immune cell infiltration degree or survival of LIHC (Fig. 4b). We also determined the infiltration degrees in LIHC showing diverse SCNA for MKI67 (Fig. 4c). According to the results obtained, MKI67 exerts a vital role in modulating the infiltration degrees of immune cells in LIHC, especially for neutrophils and DCs (Fig. 4c). This study revealed that MKI67 had a stimulating effect on the degree of immune infiltration, particularly for CD4+ T cells, neutrophils, B cells, and dendritic cells, in LIHC.

### Association of MKI67 with TIIC gene maker

We analysed the association of MKI67 levels with TIIC gene markers in LIHC based on GEPIA2 and TIMER to reveal the underlying relationship of MKI67 expression with tumour immune infiltration degree. Based on our previous study, we adopted commonly used TIIC gene markers and diverse functional T cells, including B cells, T cells, CD8+ T cells, monocytes, M1/M2 macrophages, TAMs, natural killer (NK) cells, neutrophils, mast cells, DCs, Tfhs, effector T cells, Th1/Th2/Th17 cells, central memory T cells, effector memory T cells, exhausted T cells, resident memory T cells, effector Treg T cells and resting Treg T cells. Table 2 reveals the tumour purity-adjusted results of the correlation analysis for LIHC. MKI67 was markedly related to gene markers of monocytes, B cells, M1/M2 macrophages, TAMs, CD8+ T cells, neutrophils, T cells, DCs, mast cells, NK cells, and many functional T cells, including effector memory T cells, effector T cells, resident memory T cells, central memory T cells, resting Treg T cells, exhausted T cells, Th1/Th2/Th17/Tfh cells, and effector Treg T cells. Interestingly, such observations verified that MKI67 was related to T cells, B cells, and functional T cells, as reported previously. It also illustrated the new relationship of MKI67 with exhausted T cells; however, there is little relevant research confirming this relationship.

**Table 2:** Association of MKI67 with immune cell gene markers in LIHC detected through TIMER

Immune cell	Gene markers	None		Purity	
		Cor	*P* value	Cor	*P* value
CD8+ T cell	CD8A	0.218	***	0.211	***
	CD8B	0.180	**	0.182	**
T cell	CD6	0.214	***	0.215	***
	CD3D	0.279	***	0.278	***
	CD3E	0.217	***	0.214	***
	SH2D1A	0.195	**	0.194	**
	TRAT1	0.183	**	0.196	**
	CD3G	0.279	***	0.274	***
	CD2	0.224	***	0.227	***
B cell	CD19	0.247	***	0.242	***
	FCRL2	0.202	***	0.214	***
	KIAA0125	0.146	*	0.146	*
	TNFRSF17	0.133	0.01	0.142	*
	SPIB	0.376	***	0.357	***
	PNOC	0.197	**	0.211	***
	CD79A	0.176	**	0.172	*
Monocyte	CD86	0.316	***	0.314	***
	CD115(CSF1R)	0.184	**	0.190	**
TAM	CD68	0.272	***	0.263	***
	IL10	0.249	***	0.240	***
M1 Macrophage	IRF5	0.412	***	0.411	***
	COX2(PTGS2)	0.138	*	0.151	*
M2 Macrophage	CD163	0.106	0.041	0.109	0.042
	MS4A4A	0.119	0.022	0.128	0.017
Neutrophils	FPR1	0.239	***	0.244	***
	SIGLEC5	0.296	***	0.304	***
	CSF3R	0.307	***	0.302	***
	FCGR3B	0.158	*	0.153	*
	CEACAM3	0.120	0.021	0.136	0.017
	CD116(ITGAM)	0.296	***	0.312	***
Natural killer cell	XCL2	0.210	***	0.226	***
	KIR2DL1	− 0.003	0.955	− 0.023	0.668
	KIR2DL3	0.177	**	0.189	**
	KIR2DL4	0.177	**	0.167	*
Dendritic cell	CCL13	0.181	**	0.162	*
	CD209	0.171	**	0.180	**
	HSD11B1	− 0.352	***	− 0.344	***
	HLA-DPB1	0.185	**	0.185	**
	HLA-DQB1	0.148	*	0.148	*
	HLA-DRA	0.196	**	0.199	**
	HLA-DPA1	0.176	**	0.186	**
	BCDA-1(CD1C)	0.136	*	0.141	*
	BDCA-4(NRP1)	0.291	***	0.296	***
	CD11c(ITGAX)	0.363	***	0.354	***
Mast cell	TPSB2	− 0.028	0.595	− 0.044	0.413
	HDC	− 0.166	*	− 0.175	*
Th1	IFN-γ(IFNG)	0.269	***	0.282	***
	TNF-α(TNF)	0.288	***	0.299	***
	STAT4	0.259	***	0.263	***
	STAT1	0.379	***	0.379	***
Th2	GATA3	0.250	***	0.258	***
	STAT6	0.162	*	0.157	*
	STAT5A	0.329	***	0.329	***
Tfh	BCL6	0.172	**	0.179	**
	IL21	0.172	**	0.178	**
Th17	STAT3	0.200	**	0.198	**
	IL17A	0.100	0.054	0.099	0.065
Effector T cell	CX3CR1	0.199	**	0.209	***
	FGFBP2	− 0.140	*	− 0.120	0.026
	FCGR3A	0.305	***	0.309	***
Effector memory T cell	PD-1 (PDCD1)	0.344	***	0.332	***
	DUSP4	0.324	***	0.330	***
Central memory T cell	CCR7	0.131	0.011	0.136	0.011
	SELL	0.175	**	0.188	**
	IL7R	0.176	**	0.185	**
Resident memory T cell	CD69	0.176	**	0.174	*
	ITGAE	0.306	***	0.297	***
	CXCR6	0.201	***	0.203	**
	MYADM	0.391	***	0.375	***
Exhausted T cell	TIM-3 (HAVCR2)	0.344	***	0.345	***
	TIGIT	0.325	***	0.333	***
	LAG3	0.306	***	0.295	***
	CXCL13	0.273	***	0.295	***
	LAYN	0.227	***	0.243	***
Resting Treg T cell	FOXP3	0.194	**	0.223	***
	IL2RA	0.308	***	0.309	***
Effector Treg T cell	CTLA4	0.354	***	0.353	***
	CCR8	0.444	***	0.456	***
	TNFRSF9	0.371	***	0.386	***

TAM, tumor-associated macrophage; LIHC, Liver hepatocellular carcinoma; Treg, regulatory T cells; Tfh, Follicular helper T cells; Th, T helper cells; Purity, tumor purity-adjusted correlation. None, unadjusted correlation; Cor, ρ-value upon Spearman correlation. Significance levels: * P < 0.05; ** P < 0.01; *** P < 0.001

This study also examined correlations of MKI67 levels with TIIC gene markers in LIHC and noncarcinoma tissues using GEPIA2 (Table 3) to confirm the above observations. MKI67 correlated positively with TAMs (Cor = 0.27, *P* < 0.0001), monocytes (Cor = 0.28, *P* < 0.0001), CD8+ T cells (Cor = 0.2, *P* < 0.001), neutrophils (Cor = 0.37, *P* < 0.0001), M1 macrophages (Cor = 0.31, *P* < 0.0001), and several functional T cells, particularly effector memory T cells (Cor = 0.4, *P* < 0.0001) and effector T cells (Cor = 0.26, *P* < 0.0001). Here, MKI67 was significantly related to several critical genes involved in T cell exhaustion, including TIM-3 (Cor = 0.14, *P* < 0.01), PD-1 (Cor = 0.15, *P* < 0.01), CXCL13 (Cor = 0.085, *P* < 0.1), and TIGHT (Cor = 0.21, *P* < 0.0001). These genes have an essential effect on existing antitumour immunotherapies. However, for the remaining immune cell types, such as NK cells, DCs, and neutrophils, we found statistically significant differences but a weak correlation strength compared with the above cell types. In comparison, MKI67 expression did not have a significant link with TIIC gene markers in noncarcinoma tissues.

**Table 3:** The correlations of MKI67 with immune cell gene markers for LIHC by GEPIA2

Immune cell	Gene markers	Tumor		Tumor-sum		Normal		Tumor-sum	
		Cor	*P *value	Cor	*P *value	Cor	*P *value	Cor	*P *value
CD8+ T cell	CD8A	0.2	**	0.2	**	0.39	*	0.37	*
	CD8B	0.23	***			0.31	0.026		
T cell	CD6	0.12	0.024	0.2	**	0.24	0.093	0.41	*
	CD3D	0.23	***			0.42	*		
	CD3E	0.13	*			0.34	0.017		
	SH2D1A	0.13	0.014			0.39	*		
	TRAT1	0.049	0.35			0.36	*		
	CD3G	0.24	***			0.35	0.013		
	CD2	0.15	*			0.3	0.035		
B cell	CD19	0.096	0.066	0.17	*	0.28	0.051	0.44	*
	FCRL2	0.068	0.19			0.5	**		
	KIAA0125	0.052	0.32			0.52	**		
	TNFRSF17	0.06	0.25			0.43	*		
	SPIB	0.13	0.01			0.22	0.12		
	PNOC	0.071	0.18			0.48	**		
	CD79A	0.062	0.24			0.38	*		
Monocyte	CD86	0.32	***	0.28	***	0.21	0.15	0.28	0.052
	CD115(CSF1R)	0.25	***			0.27	0.056		
TAM	CD68	0.26	***	0.27	***	0.2	0.15	0.21	0.14
	IL10	0.21	***			0.15	0.29		
M1 Macrophage	IRF5	0.31	***	0.31	***	0.13	0.37	0.07	0.63
	COX2(PTGS2)	0.078	0.14			− 0.032	0.82		
M2 Macrophage	CD163	0.15	*	0.12	0.018	0.18	0.22	0.25	0.078
	MS4A4A	0.16	*			0.22	0.12		
Neutrophils	FPR1	0.25	***	0.37	***	− 0.035	0.81	0.15	0.3
	SIGLEC5	0.31	***			− 0.011	0.94		
	CSF3R	0.22	***			0.11	0.43		
	FCGR3B	0.05	0.34			0.17	0.24		
	CEACAM3	0.19	**			0.064	0.66		
	CD116(ITGAM)	0.33	***			0.36	0.011		
CD8+ T cell	CD8A	0.2	**	0.2	**	0.39	*	0.37	*
	CD8B	0.23	***			0.31	0.026		
T cell	CD6	0.12	0.024	0.2	**	0.24	0.093	0.41	*
	CD3D	0.23	***			0.42	*		
	CD3E	0.13	*			0.34	0.017		
	SH2D1A	0.13	0.014			0.39	*		
	TRAT1	0.049	0.35			0.36	*		
	CD3G	0.24	***			0.35	0.013		
	CD2	0.15	*			0.3	0.035		
B cell	CD19	0.096	0.066	0.17	*	0.28	0.051	0.44	*
	FCRL2	0.068	0.19			0.5	**		
	KIAA0125	0.052	0.32			0.52	**		
	TNFRSF17	0.06	0.25			0.43	*		
	SPIB	0.13	0.01			0.22	0.12		
	PNOC	0.071	0.18			0.48	**		
	CD79A	0.062	0.24			0.38	*		
Monocyte	CD86	0.32	***	0.28	***	0.21	0.15	0.28	0.052
	CD115(CSF1R)	0.25	***			0.27	0.056		
TAM	CD68	0.26	***	0.27	***	0.2	0.15	0.21	0.14
	IL10	0.21	***			0.15	0.29		
M1 Macrophage	IRF5	0.31	***	0.31	***	0.13	0.37	0.07	0.63
	COX2(PTGS2)	0.078	0.14			− 0.032	0.82		
M2 Macrophage	CD163	0.15	*	0.12	0.018	0.18	0.22	0.25	0.078
	MS4A4A	0.16	*			0.22	0.12		
Neutrophils	FPR1	0.25	***	0.37	***	− 0.035	0.81	0.15	0.3
	SIGLEC5	0.31	***			− 0.011	0.94		
	CSF3R	0.22	***			0.11	0.43		
	FCGR3B	0.05	0.34			0.17	0.24		
	CEACAM3	0.19	**			0.064	0.66		
	CD116(ITGAM)	0.33	***			0.36	0.011		
Central memory T cell	CCR7	0.063	0.23	0.12	0.023	0.37	*	0.38	*
	SELL	0.1	0.055			0.24	0.092		
	IL7R	0.042	0.43			0.31	0.031		
Resident memory T cell	CD69	0.063	0.23	0.28	***	0.3	0.037	0.25	0.075
	ITGAE	0.21	***			− 0.046	0.75		
	CXCR6	0.12	0.02			0.25	0.085		
	MYADM	0.27	***			− 0.092	0.52		
Exhausted T cell	TIM-3(HAVCR2)	0.14	*	0.3	***	0.15	0.31	0.37	*
	PD-1 (PDCD1)	0.15	*			0.46	**		
	TIGIT	0.21	***			0.34	0.015		
	LAG3	0.16	*			0.15	0.31		
	CXCL13	0.085	0.1			0.28	0.049		
	LAYN	0.12	0.024			0.26	0.073		
Resting Treg T cell	FOXP3	0.011	0.84	0.18	**	0.29	0.038	0.47	**
	IL2RA	0.2	***			0.18	0.21		
Effector Treg T cell	CTLA4	0.21	***	0.27	***	0.36	*	0.4	*
	CCR8	0.23	***			0.37	*		
	TNFRSF9	0.018	0.74			0.36	*		

Cor, ρ value of Spearman's correlation. Tumor, single gene marker correlation analysis in LIHC tissue. Normal, single gene marker correlation analysis in normal tissue; Cor, ρ-value upon Spearman correlation. Significance levels: *P < 0.05; **P < 0.01; ***P < 0.001

### Expression levels of MKI67 in LIHC

**Fig. 5:**
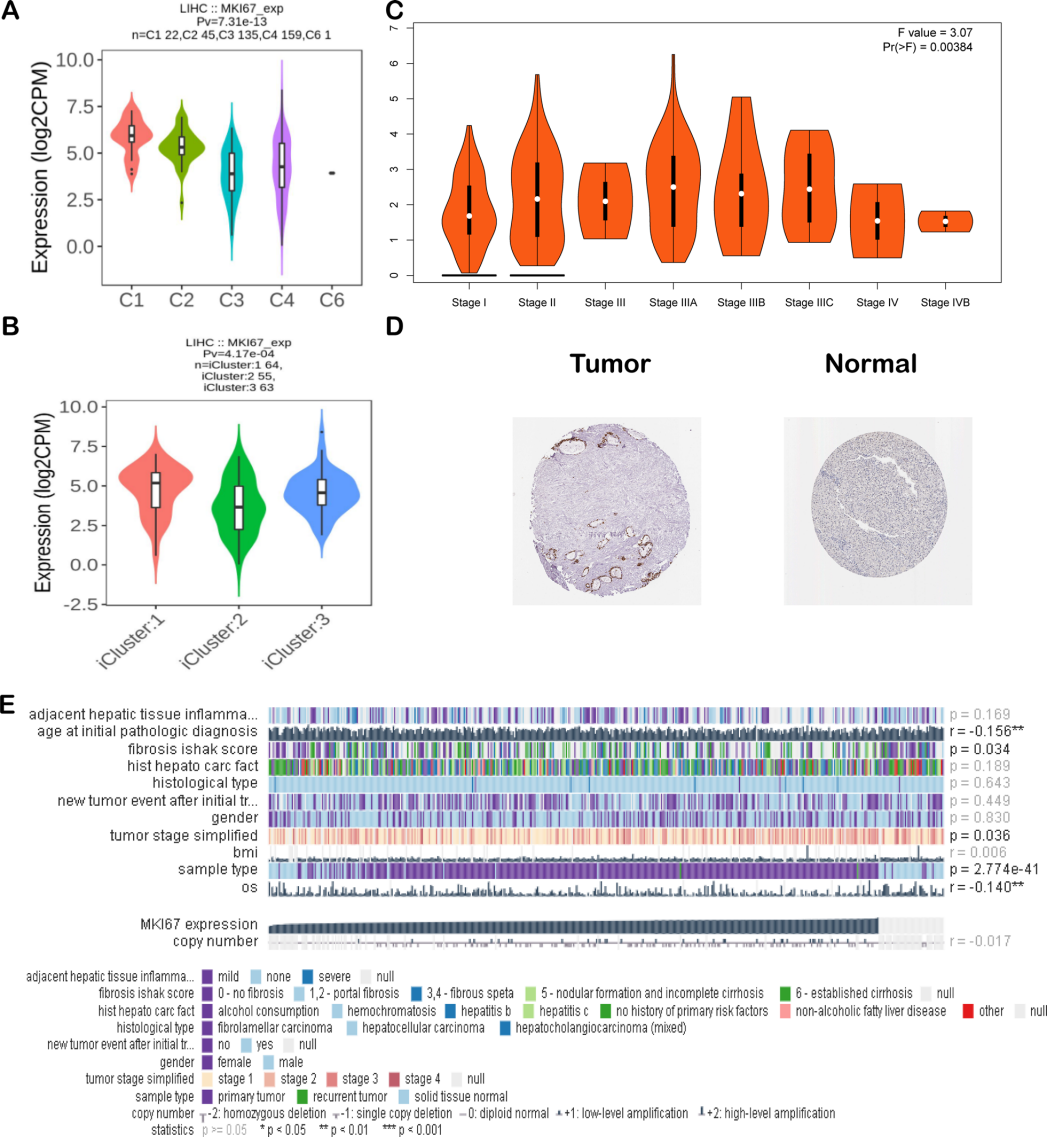
MKI67 expression levels in liver hepatocellular carcinoma. **a** MKI67 expression in TISIDB-derived LIHC having diverse molecular subtypes; **b** MKI67 expression in TISIDB-derived LIHC having diverse immune subtypes; **c** MKI67 expression in GEPIA-derived LIHC of diverse stages (MKI67 gene levels were calculated relative to the log counts per million mapped reads (log2CPM) in (**a, b**) and log2(TPM + 1) in (**c**); **d** immunohistochemistry (IHC) for MKI67 based on the HPA database; **e** MKI67 level related to PR/ER status and sample type. T: Protein levels of MKI67 in tumor tissue (staining: high; intensity: strong, quantity: 75–25%) https://www.proteinatlas.org/ENSG00000148773-MKI67/pathology/liver+cancer#img; N: Protein levels of MKI67 in normal tissue (staining: Not detected; intensity: weak, quantity: < 25%) https://www.proteinatlas.org/ENSG00000148773-MKI67/tissue/liver#img

This study also examines MKI67 levels in diverse LIHC immune subtypes in the TISIDB. As a result, we detected MKI67 expression in 4 subtypes, namely, C1 (wound healing), C2 [interferon γ (IFN-γ) dominance], C3 (inflammation), C4 (lymphocyte depletion), C5 (immunological quiet), and C6 (TGF-b dominance). The greatest MKI67 expression was detected in the C1 type, whereas the lowest was measured in the C3 type (Fig. 5a). We also measured MKI67 levels in diverse LIHC molecular subtypes (iCluster:1, iCluster:2, and iCluster:3) in TISIDB [44]. According to our results, the greatest and lowest MKI67 levels were found in the iCluster: 1 and iCluster: 2 subtypes, respectively (Fig. 5b). Furthermore, MKI67 was closely associated with the tumour immune microenvironment (TIME). Shmulevich’s work revealed that six immune subtypes were clustered for cancer [45]. According to the GEPIA database, in comparison with LIHC at diverse stages, the upregulated level was detected at stage III, while the downregulated level was detected at stages I and IV (Fig. 5c). As revealed by HPA-based analysis, more intense MKI67 staining was detected within LIHC samples than in noncarcinoma samples (Fig. 5d). In addition, according to MEXPRESS-based analysis, MKI67 levels were related to sample type, simplified tumour stage, and fibrosis risk score (Fig. 5e).

### MKI67 co-expression networks within LIHC

**Fig. 6:**
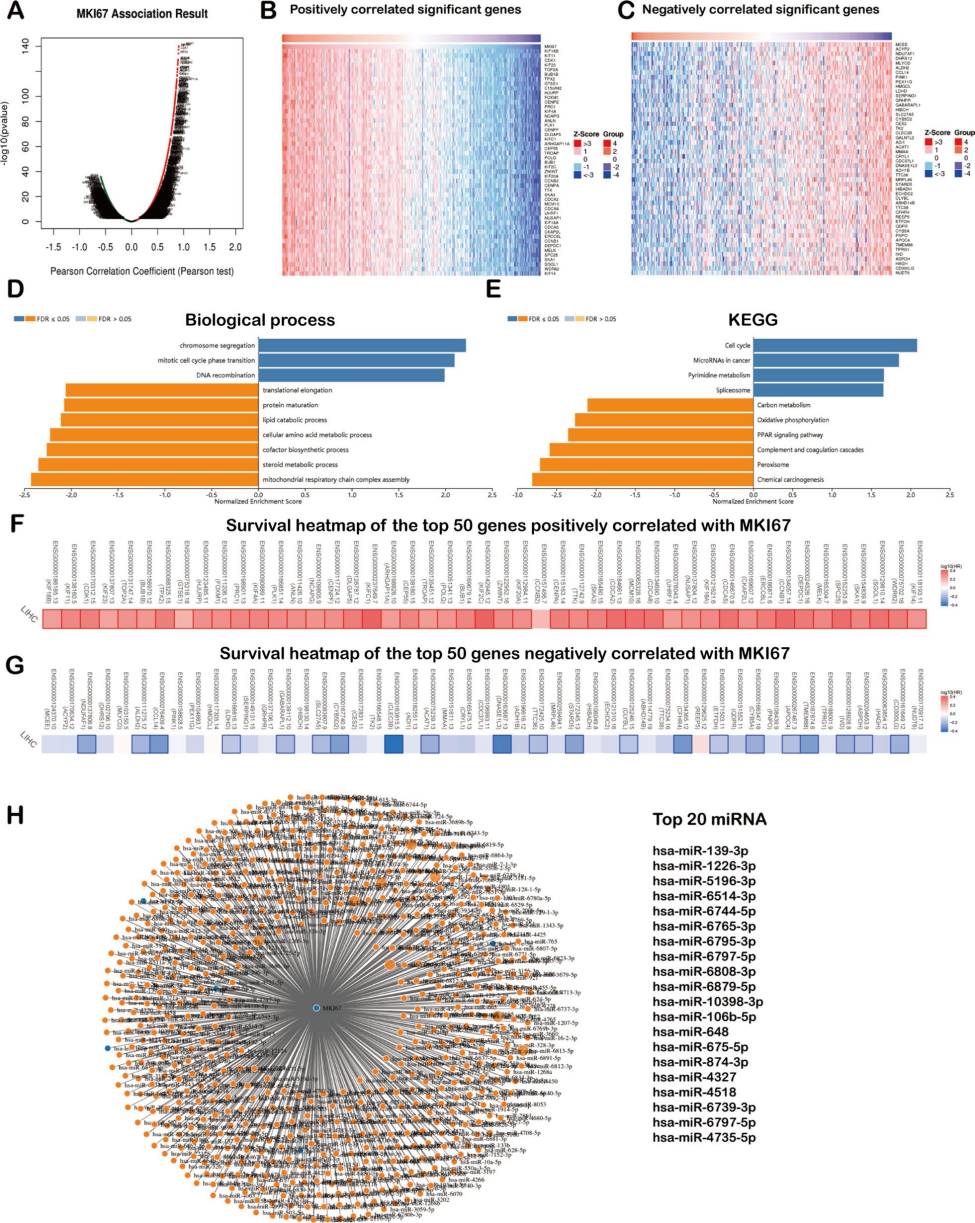
MKI67 co-expression genes in LIHC (LinkedOmics). **a** MKI67-related genes in LIHC discovered by Pearson’s test. Green and red dots indicate genes with significant negative and positive correlations with MKI67, separately; **b, c** Heatmaps displaying 50 most significant MKI67-related genes in LIHC (both positive and negative); **d, e** Significantly associated GO: BP annotations together with KEGG pathway analysis for MKI67 in LIHC; **f, g** survival heatmaps displaying the 50 most significant MKI67-related genes in LIHC (both positive and negative). Survival heatmaps displaying the log10 HRs of diverse genes. Blue and red blocks represent decreased and increased risks distinctly. Rectangles with frames represent significantly positive and negative outcomes upon prognosis analysis (*P* < 0.05). *GO* Gene Ontology, *KEGG* Kyoto Encyclopedia of Genes and Genomes, *FDR* false discovery rate, *LIHC* Liver hepatocellular carcinoma; **h** MKI67 and its predicted miRNAs (MKI67 presented in yellow circles and targeted miRNAs presented in blue circles. The interaction between the MKI67 and related miRNAs is represented in the form of lines)

To further understand MKI67’s biological significance in LIHC, LinkedOmics of the “LinkFinder” module was adopted to check the MKI67 coexpression pattern. Figure 6a reveals that 13,073 genes (red dots) showed a positive correlation with MKI67, whereas 6848 (green dots) showed a negative correlation (*P* < 0.05). Figure 6b, c display the heatmaps for the 50 most significant MKI67-related genes (both positive and negative). According to the GSEA-annotated GO terms, MKI67 coexpression genes were mainly associated with DNA recombination, chromosome segregation, and mitotic cell cycle phase transition in contrast to cellular amino acid metabolic process, translational elongation, steroid metabolic process, protein maturation, lipid catabolic process, cofactor biosynthetic process, and mitochondrial respiratory chain complex assembly (Fig. 6d). As revealed by KEGG analysis, these genes were primarily associated with the cell cycle, microRNAs in cancer, pyrimidine metabolism, spliceosomes, etc. (Fig. 6e). Notably, the 50 genes with the most significant positive correlation became high-risk markers for LIHC, among which 49 showed great HRs (HR, *P* < 0.05) (Fig. 6f). In contrast, 21 of the 50 genes with a significant negative correlation showed low HRs (*P* < 0.05) (Fig. 6g).

**Fig. 7:**
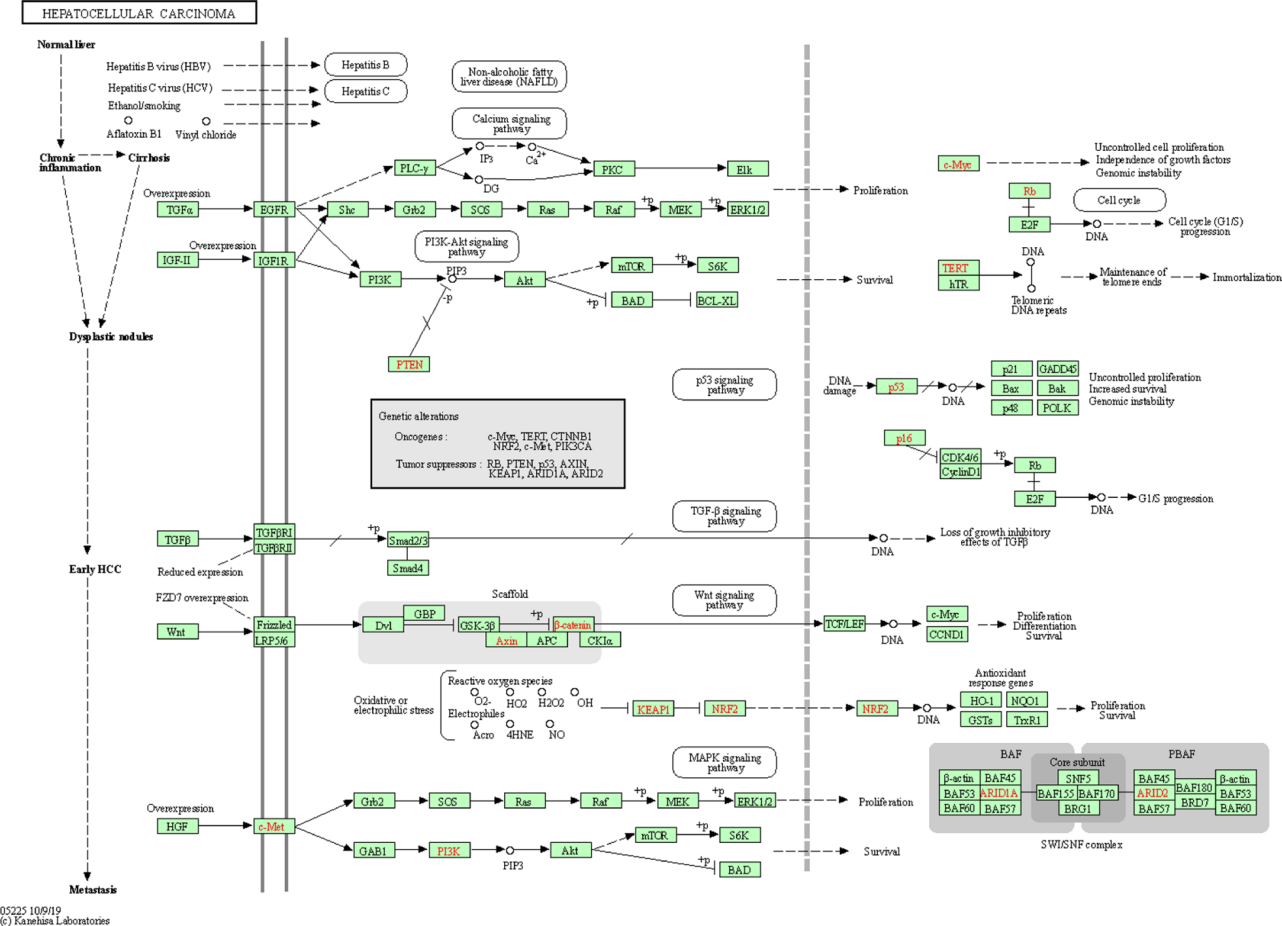
Hepatocellular carcinoma pathway regulated by the MKI67 alteration in LIHC

Among these pathways, the hsa04151:PI3K-Akt signalling pathway, hsa04115: p53 signalling pathway, hsa04010: MAPK signalling pathway, hsa04310:Wnt signalling pathway, hsa04350:TGF-beta signalling pathway, and hsa04110: cell cycle pathway were involved in the tumorigenesis and pathogenesis of liver hepatocellular carcinoma (Fig. 7).

### miRNA screening of regulatory MKI67

We applied miRWalk to screen the targeted miRNAs of MKI67. Then, we drew the miRNA-gene network using miRWalk. As illustrated in 6H, the interaction network consisted of MKI67 and 1003 miRNAs. Moreover, the contribution level of the miRNAs to MKI67 is represented as the number of lines. Additionally, the top 20 miRNAs targeting MKI67 are presented in Fig. 6h.

## Discussion

Risk factors for HCC include viral hepatitis infection, alcoholism, autoimmune diseases, and cholestasis [46–49]. The combination of FFAs and cholesterol induces severe inflammation and steatohepatitis and, at the cellular level, impairs mitochondrial function and biogenesis [50]. Recently, medical experts believe that obesity-related nonalcoholic steatohepatitis (NASH) or nonalcoholic fatty liver disease (NAFLD) is the main cause of HCC [51, 52]. Some scholars have shown that sartan significantly improved the recurrence time of liver cancer patients after rf ablation but did not improve the five-year survival rate of LIHC. Stiffness declines significantly after in hepatitis virus patients undergoing antiviral therapy [53, 54]. Plenty of evidence supports the role of MKi67 in diagnosing cancer [17–20]. MKi67, located on chromosome 10q25-ter, can encode 2 isoforms (345 and 395 kDa, respectively) [8–10]. The positive MKI67 rate (referred to as the labelling index) is suggested to be related to the clinicopathological characteristics or the survival of cancers, such as LIHC [21]. MK167 upregulation in cancer tissues is related to early cancer relapse and advanced tumour grade, according to an article enrolling LIHC cases receiving surgery [22, 23]. In addition, p53 and MK167 staining are used extensively for predicting LIHC prognosis postoperatively or even after liver transplantation [24, 25]. Such results indicate the vital role of MKI67 in tumour invasion, migration, and development. Nonetheless, the association of MKI67 levels with T cell activity, prognosis, and immune infiltration in diverse cancers remains unclear. We obtained cancer samples from some large-scale databases for analysis. According to our results, MKI67 expression is related to the survival of diverse cancers, such as LIHC. Furthermore, MKI67 coexpression genes were suggested to play a significant role in the prognosis prediction of cancer. More investigations suggest that MKI67 levels are related to the degree of immune infiltration within LIHC. Therefore, MKI67 was identified as a candidate prognostic biomarker for LIHC, which offers a new direction for understanding the association of MKI67 with T cell activity and immune infiltration.

The present study analysed MKI67 expression with prognosis expression profiles in different types of cancers based on individual datasets from Oncomine and 33 TCGA-derived cancers in GEPIA2. MKI67 expression levels in cancer and noncarcinoma samples were studied. Based on the Oncomine database, MKI67 showed high expression in bladder cancer, CNS and brain, breast cancer (BC), colorectal cancer (CRC), cervical cancer, oesophageal cancer (EC), head and neck cancer (HNC), gastric cancer (GC), liver cancer, ovarian cancer, lung cancer (LC), lymphoma, sarcoma, and pancreatic cancer compared with noncarcinoma tissues. However, according to particular datasets, MKI67 was expressed at low levels in CNS and brain cancers, BC, leukaemia, and kidney cancer (Fig. 1a). However, TCGA-based data analysis revealed high expression of MKI67 in BLCA, KIRC, BRCA, CHOL, COAD, ESCA, HNC, KICH, KIRP, LUSC, LUAD, LIHC, READ, PRAD, STAD, UCEC, and THCA and low expression within SKCM relative to noncarcinoma samples (Fig. 1b). Human Protein Atlas data further verified MKI67 upregulation in liver cancer through immunohistochemistry (Fig. 5d). Different MKI67 levels measured in diverse cancers from distinct databases can provide a novel data extraction method and help illustrate the mechanisms associated with diverse biological characteristics. However, as discovered from the above databases, MKI67 expression is related to the prognosis of LIHC, KIRP, ACC, BRCA, and LUAD. According to TCGA-based analysis, MKI67 upregulation is associated with many cancers’ dismal prognostic outcomes (PAAD, SARC, KIRP, and UVM). However, in STAD and ESCA, MKI67 downregulation predicted favourable patient survival. Concerning the GEPIA2 datasets, MKI67 upregulation might serve as a factor to independently predict the dismal survival of LIHC and ACC (Fig. 2b, d). According to the Kaplan–Meier Plotter database, MKI67 upregulation related to high HRs predicted OS and PFS for LIHC, BRCA, and LUAD (Fig. 2f, g, i). Collectively, the above results suggest the feasibility of MKI67 as a prognostic biomarker for LIHC. The present work evaluated the association of MKI67 with immunity based on the TISIDB database. According to our results, MKI67L is closely related to lymphocytes (including monocytes, type 2 helper cells, and activated CD4 T cells), immunostimulators (such as MICB, CD276, and TNFSF4), immunoinhibitors (including KDR and PDCD1 CTLA4), and MHC molecules (including B2M and HLA-C HLA-E). Therefore, MKI67 can act as a novel target to investigate immune escape in LIHC cells and a therapeutic target for anti-LIHC immunotherapy.

Liver cancer is not a single disorder but is further classified into numerous molecular subtypes. According to the TISIDB database analysis, the MKI67 gene displayed the greatest expression within the iCluster: 1 subtype, while iCluster: 3 type ranked second, whereas MKI67 was expressed at low levels in the iCluster: 2 type. Differential MKI67 expression in LIHC of diverse immune subtypes was detected. The results suggested that the C1 type displayed the most significant expression relative to those in the remaining three subtypes. Comprehensive analysis of MKI67 levels within diverse LIHC subtypes from diverse databases suggests the vital role of MKI67 in microenvironment characteristics.

Given that MKI67 has an important effect on the immune system and predicts the prognosis of LIHC, this study examined the associations of MKI67 with immune infiltration degrees within LIHC (Fig. 4a). MKI67 upregulation is closely related to the degree of immune infiltration of many immune cell subpopulations, such as B cells, neutrophils, macrophages, DCs, CD4+ T cells and CD8+ T cells (Fig. 4a). The varied SCNA for MKI67 did not significantly affect the immune infiltration degrees of CD4+ T cells or B cells within LIHC, and considerable attention was devoted to the close links between MKI67 and immune cells (Fig. 4c). According to subsequent analyses of the relationships of MKI67 with immune cell gene markers, MKI67 interacted with many immune cells and diverse functional T cells, including central memory T cells, effector T cells, and exhausted T cells (Tables 2 and 3). T cell exhaustion accounts for a leading reason for weak anticancer immunity [, and the measures for preventing T cell exhaustion represent a key role in anticancer immunotherapy [55–57]. As revealed by our results, MKI67 upregulation showed a positive correlation with several critical genes related to exhausted T cells, such as TIM-3, PD-1, LAG3, and TIGIT. These act as therapeutic targets for immunotherapy [58, 59].

Interestingly, this study suggested that the dual role of MKI67 is specific. It also predicted that MKI67 upregulation has prognostic outcomes for several cancers, including LIHC, whereas inducing T cell exhaustion shows ineffective anticancer immunity. The above two inverse trends were not contradictory. Recently, some articles have shed more light on the exact related mechanisms. Consequently, MKI67 plays a critical and distinct role in normal immunity development and regulating the TME, which is essential to identify a specific stage.

## Conclusion

The results in this work indicate the potential of MKI67 as a prognostic biomarker for several cancers, particularly LIHC. MKI67 upregulation is associated with more significant immune infiltration degrees of B cells, CD4+ T cells, CD8+ T cells, neutrophils, DCs, and many functional T cells. MKI67 exerts a stimulating effect on immunity, and it also shows a high correlation with exhausted T cells, which may serve as an essential factor to promote T cell exhaustion within LIHC. The detection of MKI67 levels possibly adds to prognosis prediction and modulation of MKI67 levels in exhausted T cells, which offers novel management for enhancing the efficacy of anti-LIHC immunotherapy.

## Supplementary Information

**Additional file 1**: **Supplementary Figure 1**. Correlation of MKI67 expression with prognostic values in diverse type cancers. **Supplementary Figure 2**. Correlation of MKI67 expression with immune infiltration level in diverse type cancers via TIMER database. **Supplementary Table 1**. Mki67 expression in cancers verus normal tissue in oncomine database.

## Data Availability

The datasets used for the current study are available from the corresponding author on reasonable request.

## Abbreviations

MKI67: Marker of proliferation Ki-67
LIHC: Liver hepatocellular carcinoma
CAM: Cell adhesion molecule
TIN: Tumor-infiltrating neutrophil
TAM: Tumor-associated macrophages
KM: Kaplan–Meier
DC: Dendritic cell
SCNA: Somatic copy number alteration
FDR: False discovery rate
FC: Fold-change
LNM: Lymph node metastasis
TME: Tumor microenvironment
