# Supplementary material for: Correlation of MKI67 with prognosis, immune infiltration, and T cell exhaustion in hepatocellular carcinoma
Source: BMC Gastroenterol. 2021 Nov 1;21:416. doi: 10.1186/s12876-021-01984-2 (PMC8561917; doi:10.1186/s12876-021-01984-2)
Supplement: Supplementary file 1 — Additional file 1: Supplementary Figure 1. Correlation of MKI67 expression with prognostic values in diverse type cancers. Supplementary Figure 2. Correlation of MKI67 expression with immune infiltration level in diverse type cancers via TIMER database. Supplementary Table 1. Mki67 expression in cancers verus normal tissue in oncomine database. [file 12876_2021_1984_MOESM1_ESM.doc]

**Supplementary Figure 1.** Correlation of MKI67 expression with prognostic values in diverse type cancers.

**
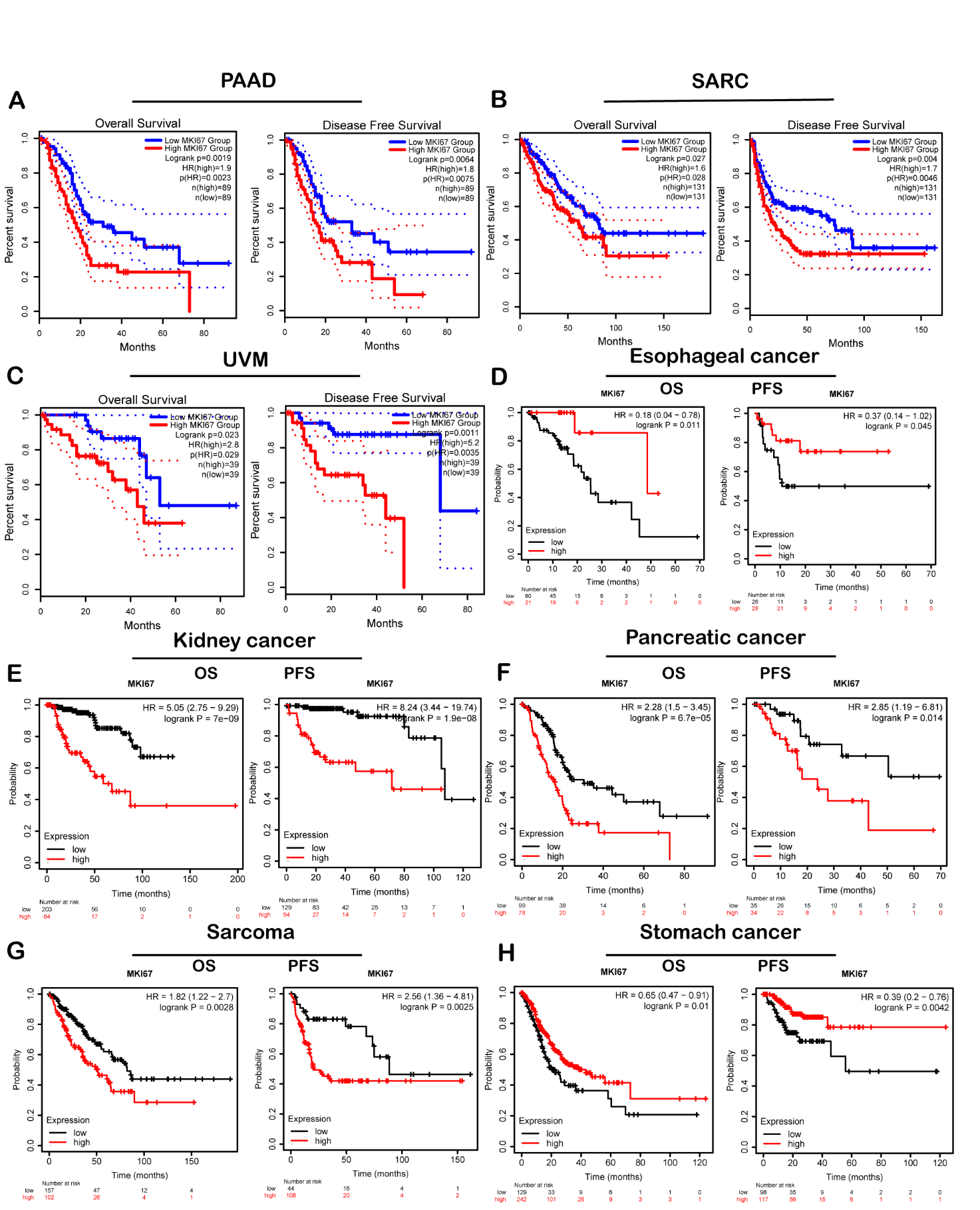
**

**Supplementary Figure 2.** Correlation of MKI67 expression with immune infiltration level in diverse type cancers via TIMER database.
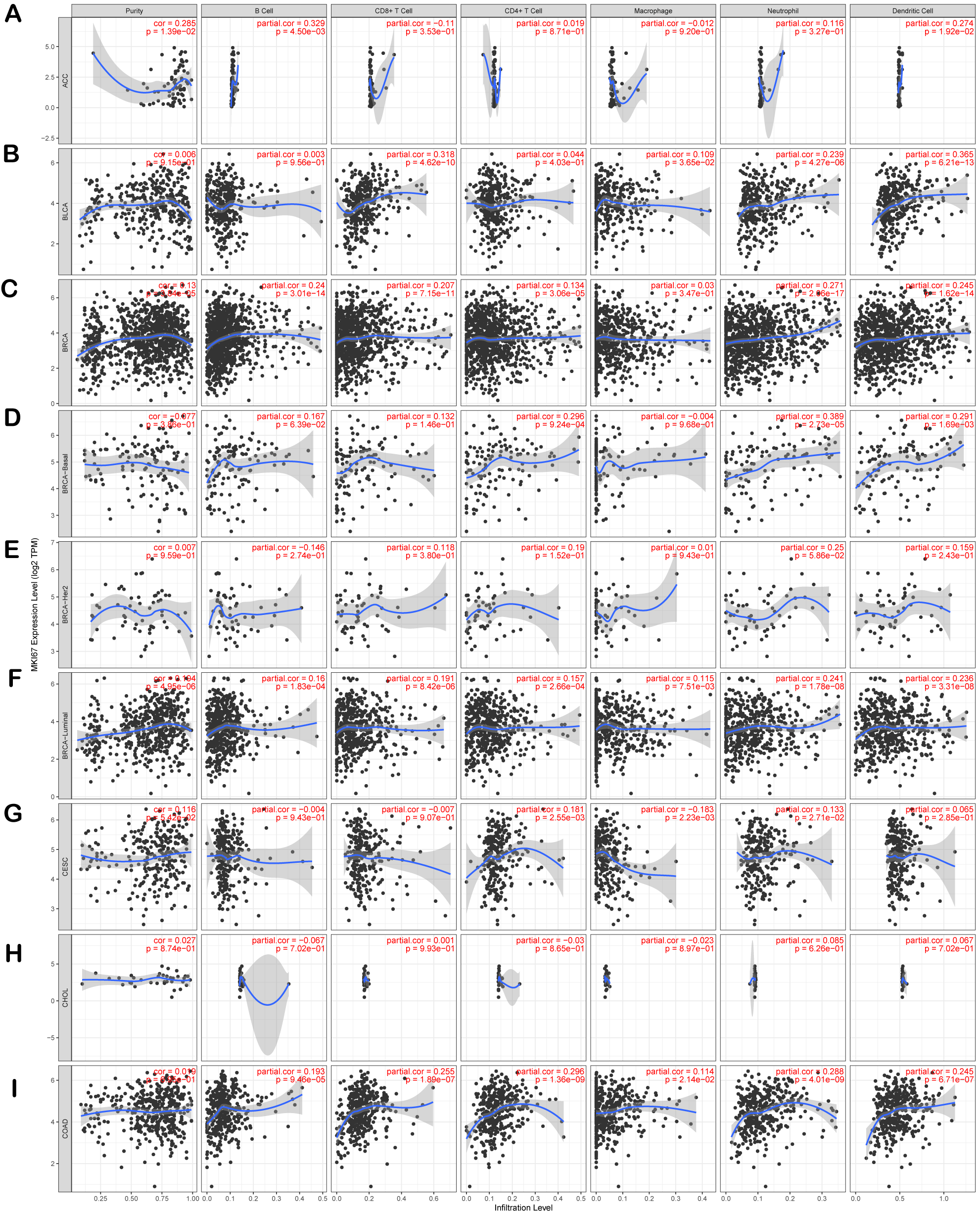


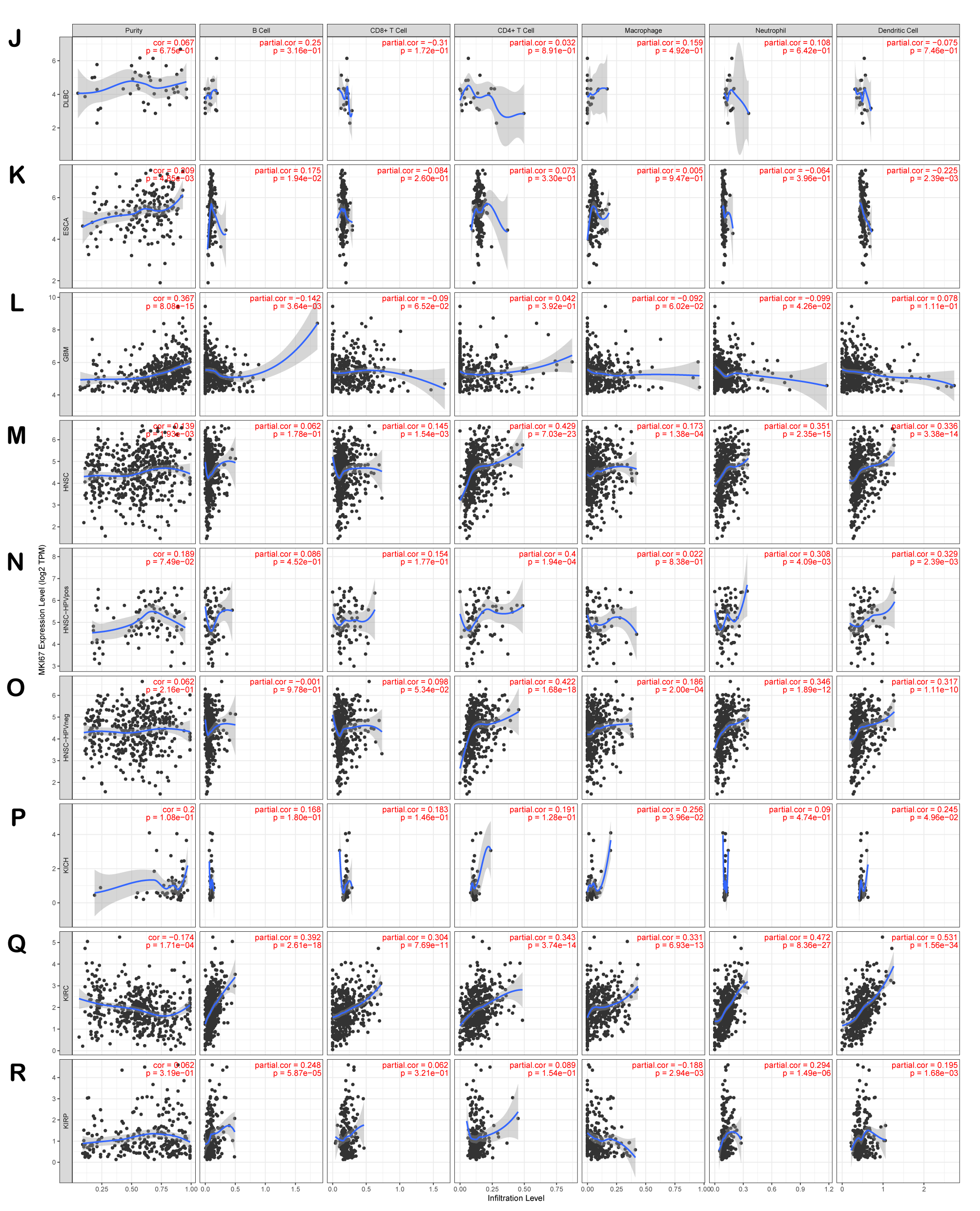


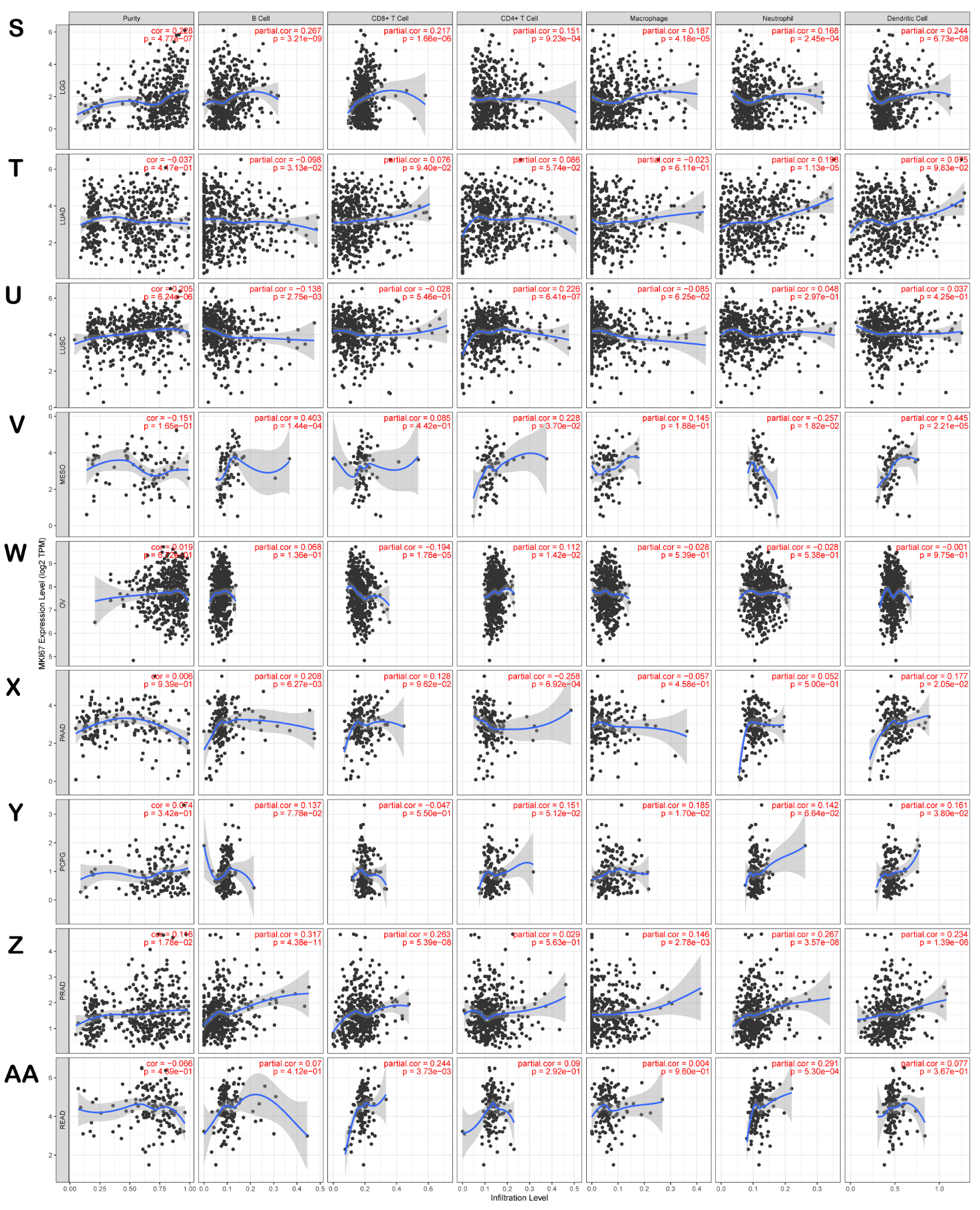


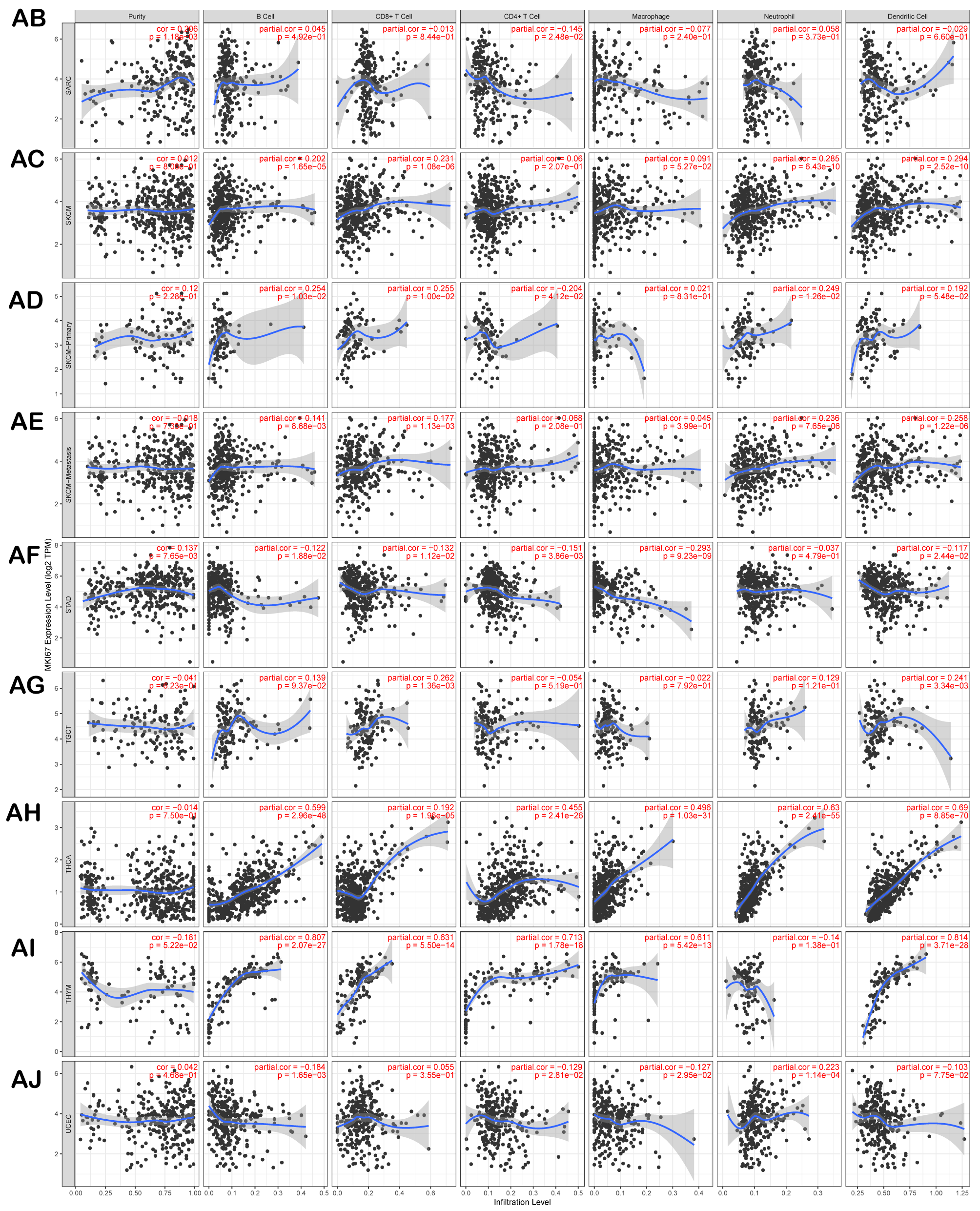


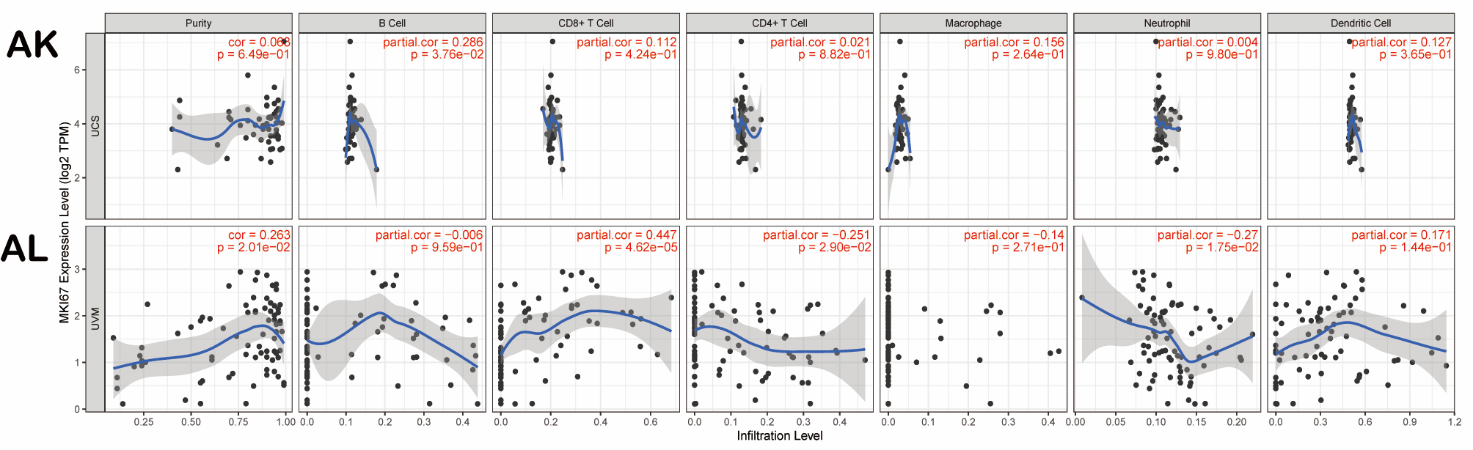


**Supplementary Table 1. Mki67 expression in cancers verus normal tissue in oncomine database**

| **Cancer** | **Cancer type** | **P-value** | **Fold**  **change** | **Rank**  **(%)** | **Sample** | **Reference**  **(PMID)** |
| --- | --- | --- | --- | --- | --- | --- |
| **Bladder cancer** | **Infiltrating Bladder Urothelial Carcinoma** | **8.20E-18** | **6.570** | **1%** | **157** | **16432078** |
|  | **Superficial Bladder Cancer** | **4.51E-15** | **4.730** | **4%** | **157** | **16432078** |
| **Brain and CNS cancer** | **Astrocytoma** | **3.50E-5** | **5.030** | **3%** | **51** | **11559565** |
|  | **Glioblastoma** | **2.29E-18** | **3.259** | **2%** | **180** | **16616334** |
|  | **Anaplastic Astrocytoma** | **3.57E-6** | **2.171** | **5%** | **180** | **16616334** |
| **Breast cancer** | **Ductal Breast Carcinoma** | **3.19E-14** | **5.601** | **1%** | **47** | **16473279** |
|  | **Invasive Breast Carcinoma** | **6.10E-32** | **5.376** | **1%** | **593** | **TCGA** |
|  | **Invasive Ductal Breast Carcinoma** | **2.70E-47** | **9.796** | **1%** | **593** | **TCGA** |
|  | **Invasive Lobular Breast Carcinoma** | **2.12E-15** | **6.261** | **2%** | **593** | **TCGA** |
|  | **Intraductal Cribriform Breast Adenocarcinoma** | **8.48E-5** | **14.072** | **4%** | **593** | **TCGA** |
| **Cervical cancer** | **Cervical Squamous Cell Carcinoma** | **1.44E-12** | **2.607** | **1%** | **66** | **18506748** |
|  | **Cervical Squamous Cell Carcinoma Epithelia** | **2.68E-8** | **2.448** | **1%** | **41** | **17974957** |
|  | **Cervical Cancer** | **3.80E-9** | **3.170** | **3%** | **84** | **17510386** |
| **Colorectal cancer** | **Rectal Adenoma** | **1.44E-6** | **3.631** | **4%** | **64** | **18171984** |
|  | **Colon Adenoma** | **3.91E-11** | **2.961** | **6%** | **64** | **18171984** |
|  | **Colon Carcinoma** | **1.11E-8** | **5.161** | **4%** | **40** | **20957034** |
|  | **Colon Carcinoma Epithelia** | **1.90E-7** | **2.945** | **5%** | **40** | **20957034** |
| **Esophageal cancer** | **Esophageal Squamous Cell Carcinoma** | **8.45E-8** | **2.068** | **2%** | **34** | **20955586** |
| **Gastric cancer** | **Gastric Cancer** | **4.42E-7** | **3.040** | **2%** | **160** | **20965966** |
|  | **Gastric Intestinal Type Adenocarcinoma** | **3.01E-5** | **2.378** | **2%** | **43** | **21701537** |
|  | **Gastric Intestinal Type Adenocarcinoma** | **4.80E-8** | **2.353** | **8%** | **69** | **19081245** |
| **Head and neck cancer** | **Tonsillar Carcinoma** | **9.62E-6** | **2.230** | **1%** | **84** | **17510386** |
|  | **Oropharyngeal Carcinoma** | **1.70E-5** | **2.366** | **2%** | **84** | **17510386** |
|  | **Tongue Squamous Cell Carcinoma** | **6.82E-6** | **2.661** | **2%** | **38** | **18254958** |
|  | **Salivary Gland Adenoid Cystic Carcinoma** | **3.66E-5** | **58.602** | **5%** | **22** | **12368205** |
|  | **Oral Cavity Squamous Cell Carcinoma** | **1.83E-12** | **2.591** | **3%** | **79** | **21853135** |
| **Liver cancer** | **Hepatocellular Carcinoma** | **2.24E-7** | **2.534** | **2%** | **75** | **17393520** |
|  | **Hepatocellular Carcinoma** | **3.70E-47** | **2.388** | **5%** | **445** | **21159642** |
| **Lung cancer** | **Lung Adenocarcinoma** | **2.17E-12** | **4.389** | **1%** | **66** | **17540040** |
|  | **Squamous Cell Lung Carcinoma** | **2.78E-25** | **6.872** | **1%** | **156** | **20421987** |
|  | **Lung Adenocarcinoma** | **5.41E-16** | **3.705** | **1%** | **156** | **20421987** |
|  | **Large Cell Lung Carcinoma** | **2.02E-8** | **4.415** | **2%** | **156** | **20421987** |
|  | **Small Cell Lung Carcinoma** | **2.35E-6** | **4.972** | **1%** | **203** | **11707567** |
|  | **Squamous Cell Lung Carcinoma** | **5.68E-6** | **6.539** | **2%** | **203** | **11707567** |
|  | **Lung Adenocarcinoma** | **1.47E-6** | **2.892** | **4%** | **39** | **16314486** |
|  | **Lung Adenocarcinoma** | **2.07E-14** | **2.498** | **3%** | **246** | **22080568** |
| **Lymphoma** | **Germinal Center B-Cell-Like Diffuse Large B-Cell Lymphoma** | **2.21E-8** | **2.330** | **3%** | **120** | **10676951** |
|  | **Activated B-Cell-Like Diffuse Large B-Cell Lymphoma** | **2.27E-6** | **2.042** | **6%** | **120** | **10676951** |
|  | **Diffuse Large B-Cell Lymphoma** | **5.87E-5** | **3.196** | **4%** | **102** | **11733578** |
|  | **Unspecified Peripheral T-Cell Lymphoma** | **7.34E-14** | **4.033** | **3%** | **60** | **17304354** |
|  | **Angioimmunoblastic T-Cell Lymphoma** | **4.37E-7** | **4.229** | **3%** | **60** | **17304354** |
|  | **Anaplastic Large Cell Lymphoma** | **1.56E-6** | **4.204** | **4%** | **60** | **17304354** |
| **Other cancer** | **Malignant Fibrous Histiocytoma** | **5.03E-8** | **11.592** | **1%** | **54** | **15994966** |
|  | **Vulvar Intraepithelial Neoplasia** | **5.48E-7** | **5.578** | **1%** | **19** | **17471573** |
|  | **Skin Basal Cell Carcinoma** | **4.80E-7** | **2.055** | **1%** | **87** | **18442402** |
|  | **Skin Squamous Cell Carcinoma** | **2.31E-5** | **3.025** | **2%** | **87** | **18442402** |
|  | **Mixed Germ Cell Tumo** | **1.58E-11** | **2.314** | **3%** | **107** | **16424014** |
|  | **Yolk Sac Tumor, NOS** | **4.15E-6** | **3.144** | **4%** | **107** | **16424014** |
|  | **Seminoma, NOS** | **3.70E-7** | **2.011** | **6%** | **107** | **16244014** |
| **Ovarian cancer** | **Ovarian Serous Cystadenocarcinoma** | **5.17E-10** | **4.023** | **1%** | **594** | **TCGA** |
|  | **Ovarian Serous Adenocarcinoma** | **1.22E-6** | **12.208** | **4%** | **53** | **19486012** |
| **Pancreatic cancer** | **Pancreatic Carcinoma** | **2.84E-11** | **2.898** | **1%** | **52** | **19732725** |
| **Sarcoma** | **Fibrosarcoma** | **4.13E-8** | **12.365** | **1%** | **54** | **15994966** |
|  | **Pleomorphic Liposarcoma** | **2.41E-7** | **10.220** | **1%** | **54** | **15994966** |
|  | **Malignant Fibrous Histiocytoma** | **5.03E-8** | **11.592** | **1%** | **54** | **15994966** |
|  | **Round Cell Liposarcoma** | **3.69E-5** | **3.391** | **2%** | **54** | **15994966** |
|  | **Myxofibrosarcoma** | **1.54E-13** | **2.690** | **1%** | **158** | **20601955** |
|  | **Pleomorphic Liposarcoma** | **2.69E-10** | **2.177** | **2%** | **158** | **20601955** |
